# Supplementary material for: COVID-19 and screen-based sedentary behaviour: Systematic review of digital screen time and metabolic syndrome in adolescents
Source: PLoS One. 2022 Mar 21;17(3):e0265560. doi: 10.1371/journal.pone.0265560 (PMC8936454; doi:10.1371/journal.pone.0265560)
Supplement: S1 Table — (PDF) [file pone.0265560.s001.pdf]

S1 Table. PRISMA 2020 Checklist

Section and Topic	Item #	Checklist item	Location where item is reported
**TITLE**			
Title	1	Identify the report as a systematic review.<br>COVID-19 and screen-based sedentary behaviour: Systematic review of digital screen time and metabolic syndrome in adolescents	Page 1
**ABSTRACT**			
Abstract	2	See the PRISMA 2020 for Abstracts checklist.<br>Checked, abstract checklist attached separately.	Page 3
**INTRODUCTION**			
Rationale	3	Describe the rationale for the review in the context of existing knowledge.<br>Prior systematic reviews and Meta-analysis have documented inconsistent association between ST and MetS among children and adolescents.<br>In light of the evolving pandemic, the prolonged screen-based sedentary behaviour exacerbated by remote learning remains a particular cause of concern. The emergence of MetS in earlier life indicates a serious risk of persistence into adulthood. Therefore, an updated evaluation of available evidence is needed to examine the association between ST and MetS (with dose-response gradient) among adolescents taking into considerations adjustment of potential confounders such as physical activity and dietary behaviour.<br>Public health measures related to the Covid-19 pandemic has led to critical increase in the use of digital screen devices and reliance on remote learning. Screen-based sedentary behaviour is linked to physical inactivity and increase caloric consumption, important contributors to obesity and cardio-metabolic risk. Taken together, a better understanding of the association between ST (of different types) and MetS among vulnerable population i.e., adolescents, is necessary to target preventable causes of premature mortality in later adulthood.	Page 5,6,16
Objectives	4	Provide an explicit statement of the objective(s) or question(s) the review addresses.<br>This systematic review aimed to summarise the findings of studies that have looked at the quantifiable association between various forms of ST and MetS in adolescents aged 12 to 18 years.	Page 6
**METHODS**			
Eligibility criteria	5	Specify the inclusion and exclusion criteria for the review and how studies were grouped for the syntheses.<br>The inclusion criteria are:<br>i. Study design: observational studies (cross-sectional, longitudinal, case-control, cohort).<br>ii. Population of interest: apparently healthy children and adolescents (12-18) year.<br>iii. Measure of ST as an exposure: Included studies that reported type of ST (TV, computer, videogames, internet, smartphone, tablet) quantified duration/frequency either self-reported or observed measure.<br>iv. Measure of MetS as an outcome: Included studies that reported standard definition and/or criteria used to establish MetS diagnosis.<br>v. Measure of relationship: examined association between ST and MetS as odds ratio (ORs) or equivalent with their 95% confidence interval (CI).<br>We excluded reviews in which ST or MetS diagnosis was not defined adequately, not an observational study design, no reporting of ORs or equivalent, studies including adolescents with pathological conditions, population younger than 12 year or older than 18 years, and studies assessing relationship of ST with outcomes other than MetS such as obesity, physical inactivity or cardiovascular risk.	Page 7-8

Section and Topic	Item #	Checklist item	Location where item is reported
Information sources	6	Specify all databases, registers, websites, organisations, reference lists and other sources searched or consulted to identify studies. Specify the date when each source was last searched or consulted.<br><br>Systematic search strategy was conducted using the following electronic bibliographic databases to identify relevant studies: PubMed Central/MEDLINE, Cochrane Library, PsycINFO and google scholar without the use of a filter to limit date of publication or language. The search was carried between August 2021 and September 2021. Only currently open access published articles were retrieved.<br>Full-text articles with reference lists were retrieved and examined for appropriateness. Another reviewer backtracked all reviewed articles for double-checking. Any discrepancies or disagreements between reviewers were resolved by either discussion or a third reviewer. Duplicate articles were removed using Reference Manager Software, and any remaining duplicates were removed manually.	Page 6-7
Search strategy	7	Present the full search strategies for all databases, registers and websites, including any filters and limits used.<br>The search was carried between August 2021 and September 2021. Only currently open access published articles were retrieved. The following keywords were used for the search: "screen time" OR "sedentary behaviour" OR "television" OR "computer" OR "internet" OR "videogames" AND "MetS" OR "cardiometabolic" OR "obesity" AND "adolescents" OR "children" OR "youth" OR "school-aged". Titles and abstracts of potentially relevant articles were screened by one reviewer to assess relevance and suitability for inclusion. Full-text articles with reference lists were retrieved and examined for appropriateness. Another reviewer backtracked all reviewed articles for double-checking. Any discrepancies or disagreements between reviewers were resolved by either discussion or a third reviewer. Duplicate articles were removed using Reference Manager Software, and any remaining duplicates were removed manually.<br>The search was done with no limitation of publication date or language.	Page 7
Selection process	8	Specify the methods used to decide whether a study met the inclusion criteria of the review, including how many reviewers screened each record and each report retrieved, whether they worked independently, and if applicable, details of automation tools used in the process.<br>Two authors (S.M., R.E.) independently screened titles and abstracts and excluded studies that were not relevant to the topic. They reviewed the full-texts of articles. First, database searches were exported into a master folder. All titles and abstracts were screened by (S.M., R.E.) and then screened by (S.M., R.E., I.D.) to assess eligibility for full-text printing and screening of references. Further, these authors independently screened all excluded titles and abstracts. If there was a disagreement, it was discussed with M.B. or N.E. to reach a final decision.<br>Not mentioned in details in the main manuscript	Page 7
Data collection process	9	Specify the methods used to collect data from reports, including how many reviewers collected data from each report, whether they worked independently, any processes for obtaining or confirming data from study investigators, and if applicable, details of automation tools used in the process.<br>Independent data extraction of studies was performed by all authors to compare data and reach consensus.<br>Titles and abstracts of potentially relevant articles were screened by one reviewer to assess relevance and suitability for inclusion<br>Cross-checking was done by other two independent authors. These reviewers backtracked all reviewed articles for double-checking. Any discrepancies or disagreements between reviewers were resolved by either discussion or a third reviewer.	Page 7
Data items	10a	List and define all outcomes for which data were sought. Specify whether all results that were compatible with each outcome domain in each study were sought (e.g., for all measures, time points, analyses), and if not, the methods used to decide which results to collect.<br>The following were extracted from each study: author, publication year, Country, Study design, sample size, mean age, gender, screen type, exposure measure (ST), outcome measure (MetS) and measure of association.	Page 11
	10b	List and define all other variables for which data were sought (e.g., participant and intervention characteristics, funding sources). Describe	NA

Section and Topic	Item #	Checklist item	Location where item is reported
		any assumptions made about any missing or unclear information.	
Study risk of bias assessment	11	Specify the methods used to assess risk of bias in the included studies, including details of the tool(s) used, how many reviewers assessed each study and whether they worked independently, and if applicable, details of automation tools used in the process.<br>The Quality Assessment Tool for Observational Cohort and Cross-sectional Studies was used to evaluate the risk of bias. The checklist comprised 14 items for longitudinal research, of which only 11 could be applied to cross-sectional studies. Each item of methodological quality was classified as yes, no, or not reported and based on number of yes as total score, studies were classified according to quality rating: Poor<50%, Fair 50-75% and good >75%. Possible disagreement on the final score were resolved by consensus among the authors. Studies met from 73% to 91% of the quality criteria, with 9 studies (9/10, 90%) meeting good scoring indicating low risk of bias. All studies clearly stated the main aim, population and definition of exposure/outcome. However, two studies (2/10, 20%) did not use key potential confounders in the analysis. Eleven items were applicable to nine studies due to cross-sectional nature of these studies and one perspective cohort study where all 14 items were applicable.	Page 8
Effect measures	12	Specify for each outcome the effect measure(s) (e.g. risk ratio, mean difference) used in the synthesis or presentation of results.<br>Results were extracted as risk estimates; Odds Ratio or Prevalence Ratio with corresponding confidence intervals or z-score of MetS. P-value of <0.05 was considered as a cut-off for statistical significance.	Page 8
Synthesis methods	13a	Describe the processes used to decide which studies were eligible for each synthesis (e.g. tabulating the study intervention characteristics and comparing against the planned groups for each synthesis (item #5)).	NA
	13b	Describe any methods required to prepare the data for presentation or synthesis, such as handling of missing summary statistics, or data conversions.	NA
	13c	Describe any methods used to tabulate or visually display results of individual studies and syntheses.	NA
	13d	Describe any methods used to synthesize results and provide a rationale for the choice(s). If meta-analysis was performed, describe the model(s), method(s) to identify the presence and extent of statistical heterogeneity, and software package(s) used.	NA
	13e	Describe any methods used to explore possible causes of heterogeneity among study results (e.g. subgroup analysis, meta-regression).	NA
	13f	Describe any sensitivity analyses conducted to assess robustness of the synthesized results.	NA
Reporting bias assessment	14	Describe any methods used to assess risk of bias due to missing results in a synthesis (arising from reporting biases).	NA
Certainty assessment	15	Describe any methods used to assess certainty (or confidence) in the body of evidence for an outcome.	NA
**RESULTS**			
Study selection	16a	Describe the results of the search and selection process, from the number of records identified in the search to the number of studies included in the review, ideally using a flow diagram.<br>PRISMA flowchart for review is presented in Figure 1	Page 10
	16b	Cite studies that might appear to meet the inclusion criteria, but which were excluded, and explain why they were excluded.	NA
Study characteristics	17	Cite each included study and present its characteristics.<br>Table 1. Summary of characteristics of included studies showing relation between screen time (ST) and Metabolic syndrome (MetS)	Page 11-12
Risk of bias in	18	Present assessments of risk of bias for each included study.	Page 9

Section and Topic	Item #	Checklist item	Location where item is reported
studies		Table (2): Study Quality Assessed by the Quality Assessment Tool for Observational Cohort and Cross-Sectional Studies	
Results of individual studies	19	For all outcomes, present, for each study: (a) summary statistics for each group (where appropriate) and (b) an effect estimate and its precision (e.g. confidence/credible interval), ideally using structured tables or plots.<br>Measure of relationship: examined association between ST and MetS as odds ratio (ORs) or equivalent with their 95% confidence interval (CI). Results were extracted as risk estimates; Odds Ratio or Prevalence Ratio with corresponding confidence intervals or z-score of MetS. P-value of <0.05 was considered as a cut-off for statistical significance.	Page 7-8
Results of syntheses	20a	For each synthesis, briefly summarise the characteristics and risk of bias among contributing studies.	NA
	20b	Present results of all statistical syntheses conducted. If meta-analysis was done, present for each the summary estimate and its precision (e.g. confidence/credible interval) and measures of statistical heterogeneity. If comparing groups, describe the direction of the effect.	NA
	20c	Present results of all investigations of possible causes of heterogeneity among study results.	NA
	20d	Present results of all sensitivity analyses conducted to assess the robustness of the synthesized results.	NA
Reporting biases	21	Present assessments of risk of bias due to missing results (arising from reporting biases) for each synthesis assessed.	NA
Certainty of evidence	22	Present assessments of certainty (or confidence) in the body of evidence for each outcome assessed.	NA
**DISCUSSION**			
Discussion	23a	Provide a general interpretation of the results in the context of other evidence.	Page 17-18
	23b	Discuss any limitations of the evidence included in the review.<br>The evidence of this review was dependent on peer-reviewed journals via scientific databases, not accessing data of unpublished reports from educational institutions, non-profit data, or community services, and maybe subject to publication bias. Due to apparent high risk of bias (ROB) attributable to clinical and methodological heterogeneity, meta-analysis of data was not performed. Moreover, undefined information in any of the included studies was not confirmed with related authors jeopardizing their quality, if any important details were missed. Furthermore, our research was limited to studies published only in English and the cross-sectional design of the majority of included studies prevented inference of causality, thereby limiting the conclusion drawn regarding the temporal relationship between ST and MetS	Page 19
	23c	Discuss any limitations of the review processes used.<br>Data were extracted by one researcher, and although data were crosschecked carefully back to the publication by the second researcher, we did not use dual independent extraction. Because this was a rapid review, we did not attempt to contact authors of any non-retrievable articles. In our narrative synthesis of findings, we aimed to avoid vote-counting of numbers of positive or negative studies to judge strength of evidence. However, it is possible that our findings reflect methodological or conceptual biases in our included reviews. Finally, the search did not extend to all existing databases. Nonetheless, we performed searches in two primary databases and one secondary database. In addition, we conducted a thorough search of all the references of the studies included in the review to find further studies.	Page 19,20
	23d	Discuss implications of the results for practice, policy, and future research.<br>The COVID-19 pandemic has resulted in children and adolescents spending more time on digital screen devices, which may have profound effect on their cardio-metabolic health. In summary, our review demonstrated that independent of physical activity, significant association between ST and MetS was noted among adolescents. This observation has important public health and clinical implications that demand urgent prevention initiatives targeting young people and their parents. Such interventions aim to increase awareness of potential adverse health impacts of ST, considering the dose-response relationship as well as the international recommendations of ST and physical activity across different age groups. Additional higher quality studies, including longitudinal and RCTs are needed to confirm this primarily observational evidence.	Page 20

Section and Topic	Item #	Checklist item	Location where item is reported
**OTHER INFORMATION**			
Registration and protocol	24a	Provide registration information for the review, including register name and registration number, or state that the review was not registered.<br>Name: Dr. Rowaida Elyamani<br>PROSPERO 2021 Registration Number: CRD42021272436.	Page 2
	24b	Indicate where the review protocol can be accessed, or state that a protocol was not prepared.	POSPERO
	24c	Describe and explain any amendments to information provided at registration or in the protocol.<br>Following submission of SR at PROSPERO, further information was requested by reviewer such as details of the sources searched, any restrictions applicable as well as requesting of information in relation to data synthesis.<br>All above amendment requests were addressed and stratified with registration. Data synthesis and Meta-analysis of this article was not performed and clearly explained.<br>Further details related to reviewer feedback and amendments process can be accessed from Dr. Rowaida Elyamani	PROSPERO<br>Dr. ROWAIDA ALYAMANI
Support	25	Describe sources of financial or non-financial support for the review, and the role of the funders or sponsors in the review.<br>No external funding related to the project has been received. Open Access funding is provided by the Qatar National Library.	Page 20
Competing interests	26	Declare any competing interests of review authors.<br>The authors declare that they have no competing interests. The authors alone are responsible for the content and writing of this article.	Page 20
Availability of data, code and other materials	27	Report which of the following are publicly available and where they can be found: template data collection forms; data extracted from included studies; data used for all analyses; analytic code; any other materials used in the review.	NA

From: Page MJ, McKenzie JE, Bossuyt PM, Boutron I, Hoffmann TC, Mulrow CD, et al. The PRISMA 2020 statement: an updated guideline for reporting systematic reviews. BMJ 2021;372:n71. doi: 10.1136/bmj.n71

For more information, visit: http://www.prisma-statement.org/
