# Supplementary material for: COVID-19 and screen-based sedentary behaviour: Systematic review of digital screen time and metabolic syndrome in adolescents
Source: PLoS One. 2022 Mar 21;17(3):e0265560. doi: 10.1371/journal.pone.0265560 (PMC8936454; doi:10.1371/journal.pone.0265560)
Supplement: S2 Table — (PDF) [file pone.0265560.s002.pdf]

S2 Table: NIH Quality Assessment Tool for Observational Cohort and Cross-Sectional Studies

The National Institutes of Health (NIH) Quality Assessment Tool was used for observational cohort and cross-sectional studies. All the studies were assigned a yes, no, or other to each of the 14 criteria outlined in the appraisal tool. Then by considering each criterion, the investigators evaluated the overall quality of the study and assigned an overall good, fair, poor rating to each study.

**Criteria**	**Yes**	**No**	**Other (CD, NA, NR)***
1. Was the research question or objective in this paper clearly stated?			
2. Was the study population clearly specified and defined?			
3. Was the participation rate of eligible persons at least 50%?			
4. Were all the subjects selected or recruited from the same or similar populations (including the same time period)? Were inclusion and exclusion criteria for being in the study prespecified and applied uniformly to all participants?			
5. Was a sample size justification, power description, or variance and effect estimates provided?			
6. For the analyses in this paper, were the exposure(s) of interest measured prior to the outcome(s) being measured?			
7. Was the timeframe sufficient so that one could reasonably expect to see an association between exposure and outcome if it existed?			
8. For exposures that can vary in amount or level, did the study examine different levels of the exposure as related to the outcome (e.g., categories of exposure, or exposure measured as continuous variable)?			
9. Were the exposure measures (independent variables) clearly defined, valid, reliable, and implemented consistently across all study participants?			
10. Was the exposure(s) assessed more than once over time?			
11. Were the outcome measures (dependent variables) clearly defined, valid, reliable, and implemented consistently across all study participants?			
12. Were the outcome assessors blinded to the exposure status of participants?			
13. Was loss to follow-up after baseline 20% or less?			

14. Were key potential confounding variables measured and adjusted statistically for their impact on the relationship between exposure(s) and outcome(s)?			

*CD, cannot determine; NA, not applicable; NR, not reported
