# Supplementary material for: COVID-19 and screen-based sedentary behaviour: Systematic review of digital screen time and metabolic syndrome in adolescents
Source: PLoS One. 2022 Mar 21;17(3):e0265560. doi: 10.1371/journal.pone.0265560 (PMC8936454; doi:10.1371/journal.pone.0265560)
Supplement: Attachment — Submitted filename: Response to Reviewers.docx [file pone.0265560.s003.docx]

**Dear Academic Editor of “PLOS ONE”**

Thank you for providing us this opportunity to further revise our manuscript entitled: **“COVID-19 and screen-based sedentary behaviour: Systematic review of digital screen time and metabolic syndrome in adolescent”** (Manuscript Number: PONE-D-21-30881).

We appreciate the very positive and constructive feedback. All needed changes were brought to this manuscript taking into account the comments of Academic Editor and the two reviewers.

Additionally, we made some extra changes that weren’t highlighted by the reviewers to provide further information or improve the academic English.

**Data Availability Statement:** All relevant data are within the manuscript and its supporting information files.

**Funding Statement:** The publication of this article was funded by Qatar National Library. The funders had no role in study design, data collection and analysis, decision to publish, or preparation of the manuscript.

We believe that we had addressed all the concerns raised by the reviewers and hope the manuscript is now acceptable to PLOS ONE.

Sincerely yours,

Number of words: 5932

Number of references: 73

**TO ACADEMIC EDITOR**

**Dear Reviewer,**

Thank you for your comments. Please find below the responses to your questions/suggestions. Sincerely yours.

| **N°** | **REMARK** | **RESPONSE** | **CORRECTIVE ACTIONS** |
| --- | --- | --- | --- |
| **1.** | Thank you for submitting your manuscript to PLOS ONE. After careful consideration, we feel that it has merit but does not fully meet PLOS ONE’s publication criteria as it currently stands. Therefore, we invite you to submit a revised version of the manuscript that addresses the points raised during the review process. | Thank you for the valuable review that will indeed strengthen our manuscript, we do appreciate the chance given.  All efforts were made to address remarks specified by the Academic Editor and the two Reviewers in order to fulfil PLOS publication criteria. | |
| **2.** | Please ensure that your manuscript meets PLOS ONE's style requirements, including those for file naming. | Thank you for providing the guideline template  We have considered all requirements based on PLOS ONE formatting guidelines. Files naming were made accordingly. | Resubmitted file names:   1. *Manuscript* 2. *Revised Manuscript with Track Changes* 3. *Response to Reviewers* |
| **3.** | Please ensure that your submission is free of typos and grammatical errors. *Please ensure that your PRISMA flowchart details the reasons for record exclusion at each step. | As instructed, we have made further grammatical, and spelling check by native speaker.  Reasons for exclusion criteria at each step are now added to the PRISMA flowchart  (Figure 1) | Figure 1. PRISMA flowchart for review, was modified ***L239-241***  The following reasons for record exclusion were added:  *Study design= 8*  *Age group= 6*  *MetS not defined adequately= 4*  *MetS not linked with ST exposure= 7*  *Non-quantifiable ST= 3*  *ST linked to outcome other than MetS= 9*  *No measure of association= 6*  *Full-text not available= 10* |
| **4.** | We note that you have provided additional information within the Acknowledgements Section that is not currently declared in your Funding Statement. Please note that funding information should not appear in the Acknowledgments section or other areas of your manuscript. We will only publish funding information present in the Funding Statement section of the online submission form.  Please remove any funding-related text from the manuscript and let us know how you would like to update your Funding Statement. Currently, your Funding Statement reads as follows:  The author(s) received no specific funding for this work.  Please include your amended statements within your cover letter; we will change the online submission form on your behalf. | Thank you for clarification.  Can we add below Funding Statement please:  The publication of this article was funded by Qatar National Library. The funders had no role in study design, data collection and analysis, decision to publish, or preparation of the manuscript. | Funding Statement was removed from the manuscript  The following statement was added to the cover letter:  *The publication of this article was funded by Qatar National Library. The funders had no role in study design, data collection and analysis, decision to publish, or preparation of the manuscript.* |
| **5.** | Please note that in order to use the direct billing option the corresponding author must be affiliated with the chosen institute. Please either amend your manuscript to change the affiliation or corresponding author or email us at plosone@plos.org with a request to remove this option. | Thank you for clarification.  We have emailed [plosone@plos.org](mailto:plosone@plos.org) and request to remove the direct billing option. | *Email sent to* [*plosone@plose.org*](mailto:plosone@plose.org)  *No changes to affiliation or corresponding author are applicable.*  *Kindly to remove the direct billing option* |
| **6.** | We note that you have stated that you will provide repository information for your data at acceptance. Should your manuscript be accepted for publication, we will hold it until you provide the relevant accession numbers or DOIs necessary to access your data. If you wish to make changes to your Data Availability statement, please describe these changes in your cover letter and we will update your Data Availability statement to reflect the information you provide. | Thank you for explaining.  Data Availability Statement was amended as the following:  All relevant data are within the manuscript and its Supporting information files. | Data Availability Statement was updated in the cover letter as the following:  ***Data Availability Statement:*** *All relevant data are within the manuscript and its Supporting information files.* |
| **7.** | Please include captions for your Supporting Information files at the end of your manuscript, and update any in-text citations to match accordingly. | Supporting information files were added to the manuscript as well as details and in-text citations. | The following Supporting Information files were added at the end of the manuscript **L710-711**  ***Supporting information***  *S1 Table. PRISMA 2020 Checklist.*  *S2 Table. NIH Quality Assessment Tool for Observational Cohort and Cross-Sectional Studies.* |
| **8.** | We noticed you have some minor occurrence of overlapping text with the following previous publication(s), which needs to be addressed:  In your revision ensure you cite all your sources (including your own works), and quote or rephrase any duplicated text outside the methods section. Further consideration is dependent on these concerns being addressed. | Thank you for your precise observations.  Kindly note that rephrasing and citation of the texts from the listed publications were performed. (details of each are found below) | |
|  | https://pubmed.ncbi.nlm.nih.gov/27997601/ | This article is under the reference number [31] | **(L132-135)**  *For instance, de Oliveira RG et al. [31] revealed that ST of more than 2h/day during the weekend was significantly associated with a twofold increased risk of MetS, while insignificant association was observed concerning other days of the week.* |
|  | https://bmjopen.bmj.com/content/bmjopen/9/1/e023191.full.pdf?ck_subscriber_id=31674 | This article is under the reference number [33] | (**L137-140)**  *Stiglic et al. [33] in their review found inadequate and unreliable evidence of association between ST and MetS, though, higher levels of ST were related significantly to an increased caloric intake, lower nutritional food, being obese, and having an overall reduced quality of life.* |
|  | https://ijbnpa.biomedcentral.com/articles/10.1186/1479-5868-8-98 | This article is under the reference number [44] | (**L345-349)**  These findings are consistent with Trenblay et al. [44], who reported in their systematic review and meta-analysis of 232 publications a positive correlation between increased levels of sedentary behaviour (especially TV viewing> 2h/day) and cardiometabolic illnesses among children and youth. A notable link was observed between ST and adverse body composition, poor fitness, and low self-esteem [44]. |
|  | https://link.springer.com/article/10.1186/s12966-018-0726-9 | This article is under the reference number [71] | **(L406-409)**  *School could be an ideal setting for encouraging physical activity and reducing sedentary behaviour. For instance, Verlogine et al. [71] acclaimed the placing of standing desks in classrooms and promoting stand-up bouts as an interventional approach to increase pupils’ self-efficacy and reduce sedentary behaviour in a pleasurable manner.* |

**TO REVIEWER #1**

**Dear Reviewer,**

Thank you for your comments. Please find below the responses to your questions/suggestions. Sincerely yours.

| **N°** | **REMARK** | **RESPONSE** | **CORRECTIVE ACTIONS** |
| --- | --- | --- | --- |
| **1.** | There are major revisions needed in the manuscript. | Thank you for your remark.  All your suggestions have been taken into considerations and changes made to full text accordingly. | |
| **2.** | The introduction should be written in more detail and clearly. | Thank you for your remark  We ado agree that a brief introduction section is advantageous to provide more context.  Changes have been done to the introduction part of the main text according to your suggestion. | *The following sentences were added:*  ***(L80-84)***  *Given the revolutionary advances in digital technologies, the question of how to adequately classify ST remains a challenge [10]. The World Health Organization (WHO) defines ST as “Time spent passively watching screen-based entertainment (TV, computers, mobile devices)," excluding other innovative and modern forms of virtual realities, interactive video-gaming where physical activity or movement is required [11]*  ***(L85-93)***  *The COVID-19 pandemic has caused a marked increase in ST across the globe. A large observational study (n=8395) in 10 European countries revealed that 69.5% [95%CI: 68.5- 70.5] of young adolescents aged 6-18 years have exceeded the recommended limit of ST (>2 h/day) during weekdays and 63.8% during weekend [95%CI: 62.7- 64.8]. Children residing in mildly affected countries and those in countries with lower level of restrictions were less likely to exceed that limit (OR= 3.25 [95%CI: 2.38 - 4.45) and OR= 1.42 [95% CI: 1.07-1.90], respectively) [12].* *Similarly, findings from (ABCD) study during the early stages of the pandemic reported a mean (SD) of 7.70 (5.74) h/day of screen use, a more than twofold increase as compared to the pre-pandemic figure* *[13].*  ***(L98-109)***  *According to the American Heart Association (AHA), National Heart, Lung and Blood Institute (NHLBI) and International Diabetes Federation (IDF), the diagnosis of MetS is based on the presence of three of the followings: waist circumference (WC) indicative of central obesity (at least 102 cm in men and 89 cm in women), raised triglyceride (<40 mg/dl) in males, <50 mg/dl in females), raised blood pressure (systolic BP≥ 130 or diastolic BP ≥ 85 mmHg or receiving treatment for hypertension), and raised fasting glucose level (≥100 mg/dL, or diagnosed with type 2 diabetes) [16]. The diagnosis of MetS is usually established after the age of 10 years. In older children and adolescents aged 10-16 years, MetS is diagnosed in the presence of central adiposity (≥90th) and two of the following: triglycerides (TG)≥ 150 mg/dl, HDL-C <40 mg/dl, systolic blood pressure (SBP) ≥ 130 mmHg or diastolic blood pressure (DBP) ≥85 mmHg, fasting plasma glucose (FG) ≥ 100 mg/dl or previously diagnosed type 2 diabetes [17].*  ***(L110-112)***  *MetS in children and adolescents has become a major public health concern with prevalence reaching as high as 38.9% in the general population and relatively higher in overweight/obese children [18].*  ***(L114-120)***  *The pathological process underlying MetS begins already in childhood with complex interrelated genetic and environmental factors [22]. Evidence suggests that screen-based behaviours and physical inactivity are associated with higher levels of inflammatory biomarkers such as interleukin-6 (IL-6) and tumour necrosis factor-α (TNF-α) which stimulate C-reactive protein (CRP), an important causative pathway leading to dyslipidaemia, insulin resistance and cardiovascular diseases [23]. A study by Strizich et al., showed that lower levels of MVPA were associated with higher glucose/lipid profile, and increased inflammatory biomarkers [24].*  ***(L126-131)***  *The association between ST and MetS among adolescents has been investigated in several studies prior to the declaration of the COVID-19 pandemic [26-27]. However, results were found inconclusive for the most part owing to limited data and generalizability of findings to different types of ST considering the duration, content, and context of exposure [28-29]. In a recent systematic review, authors pointed out limitations in approving the direct cause and effect relationship between excessive ST and MetS in adolescents [30].* |
| **3.** | The method should be better communicated to readers. Statistical analyzes should be expressed more clearly. | Thank you for highlighting this point.  We’ve added a section entitled (Data analysis) to the main text. | *The following section was added:*  ***(L223-232)***  ***Data analysis***  *Synthesis began by summarizing review results and conclusions in note form. Reviews were then grouped by the exposure, which is screen time, and the outcome of interest was measured, which is the MetS and related risk factors. Moreover, we examined the conclusions of the included studies to decide which article came out as plausible. However, we did not enumerate the findings across studies as quantitative summaries should be undertaken at an individual study level rather than at a review level. A descriptive analysis of each included publication was conducted. ST exposure in hrs/day or week and the observed prevalence of MetS in percentages were specified. Adjusted estimates of OR or MetS z-score for the association between ST and MetS with a corresponding 95% CI were obtained. The OR of the dose-response gradient effect was also extracted.* |
| **4.** | Expand the discussion section and provide advice for practical practitioners. | Thank you for the constructive recommendations. As acknowledged by the reviewer, changes have been done to the main text according to your suggestion.  Implications of our review on home, school and clinical practice were added. | *The following details were added:*  ***(L349-354)***  *Contrarily, some scholars have argued that ST in adolescents is irrelevant to future health risks. A systematic review [31] showed weak evidence of an association between ST and poor cardiorespiratory fitness, poorer cognitive development, lower educational attainments, poor sleep outcomes, or risk of MeTs. However, it is important to note that weak association does not imply absence of correlation. For instance, the authors have reported a lack of literature as the probable cause of such an observation.*  ***(L401-428)***  *Evidence is validating the role of opportunistic behaviour change counselling by clinical practitioners in the management of sedentary behaviour. A key mitigative strategy is to identify correlates of sedentary time, such as sociodemographic attributes, accessibility, parental behaviour, psychological, PA, and dietary behaviour [70]. Engaging adolescents, families, schools, and social workers in healthy lifestyle choices provides an enabling environment that supports behavioural change. School could be an ideal setting for encouraging physical activity and reducing sedentary behaviour. For instance, Verlogine et al. [71] acclaimed the placing of standing desks in classrooms and promoting stand-up bouts as an interventional approach to increase pupils’ self-efficacy and reduce sedentary behaviour in a pleasurable manner.*  *It may be possible to use a holistic method that looks at all three levels of the socio-ecological model (intra-individual, inter-individual, physical environment, and policy) to help people move more and make healthy food choices [72]. Effective communication with adolescents and their families enhances digital literacy related to screen types, content, and setting screen limits. A systematic review and meta-analysis on the effect of behavioural interventions in reducing ST revealed that smaller sample sizes and shorter intervention durations were associated with greater impact. Involvement of healthcare professionals in setting goals, feedback, and planning clusters yielded better outcomes in ST reduction [73].*  *Healthcare workers are advised to initiate early prevention strategies tackling the associated risk factors of NCDs among adolescents. Given the challenging period of COVID-19, it has become increasingly important to integrate lifestyle education, health promotion, and community awareness within the management. Screening for early identification of behavioural and metabolic risk factors will help reduce the burden of NCDs later in adulthood. Tools such as screening of baseline PA, ST, Body Mass Index (BMI) and psychological assessment are essential to identify modifiable risk factors and ‘at risk’ children. Accordingly, replacement strategies, weight loss programs, and exercise prescriptions are recommended, taking into account the importance of continuous monitoring and evaluation. Evidence from this study can guide national and public health efforts in planning accessible multisectoral prevention and intervention strategies to tackle the determinants of MetS and NCDs.* |

**TO REVIEWER #2**

**Dear Reviewer,**

Thank you for your comments. Please find below the responses to your questions/suggestions. Sincerely yours.

| **N°** | **REMARK** | **RESPONSE** | **CORRECTIVE ACTIONS** |
| --- | --- | --- | --- |
| **1.** | This review is topical and will add to the evidence to support the need for interventions to reduce sedentary behaviour across the age spectrum. The authors have presented recent data to improve research in an important area. | Thank you for your positive feedback. We do appreciate your time and efforts reviewing our manuscript. | |
